# Targeted genome sequencing data of young women breast cancer patients in Cipto Mangunkusumo national hospital, Jakarta

**DOI:** 10.1016/j.dib.2020.106138

**Published:** 2020-08-06

**Authors:** Sonar Soni Panigoro, Kristina Maria Siswiandari, Rafika Indah Paramita, Fadilah Fadilah, Linda Erlina

**Affiliations:** a Surgical Oncology Division, Department of Surgery, Faculty of Medicine, Universitas Indonesia, Jalan Salemba Raya number 6, Jakarta 10430, Indonesia; b Department of Medical Chemistry, Faculty of Medicine, Universitas Indonesia, Jalan Salemba Raya number 4, Jakarta 10430, Indonesia; c Bioinformatics Core Facilities—IMERI, Faculty of Medicine, Universitas Indonesia, Jalan Salemba Raya number 6, Jakarta 10430, Indonesia

**Keywords:** Breast cancer, Cipto mangunkusumo national hospital jakarta, Targeted-genome sequencing, Young women

## Abstract

Breast cancer is the most common cancer in women, accounting for approximately 25% of all cancer cases worldwide. Some breast cancer patients are genetically predisposed to genes involved in genomic stability. We report the targeted genome sequencing data of 24 young women (aged below 45 years) breast cancer patients admitted to Cipto Mangunkusumo National Hospital, Jakarta, Indonesia. These data will be useful in detecting the genome markers of breast cancer and in deciding the diagnostics and therapies. DNA sequences were obtained using the Illumina NextSeq 500 platform. FASTQ raw files are available under BioProject accession number PRJNA606794 and Sequence Read Archive accession numbers SRR11774092–SRR11774115.

Specifications table

**Table:** 

Subject	Human targeted genome sequencing
Specific subject area	Genomics
Type of data	Genome sequences (Targeted DNA-Seq raw reads)
Data retrieval	Illumina NextSeq 500 sequencing platform
Data format	Raw sequences (FASTQ)
Parameters for data collection	DNA was extracted from the buffy coat of whole blood sample and was quantified, followed by preparation and quality check of the DNA libraries.
Description of data collection	DNA was extracted using QIAamp DNA Mini Kit® (Qiagen Sciences, Germantown, Maryland). Double-stranded DNA concentration was quantified with a Qubit® 3.0 Fluorometer (Thermo Fisher Scientific, Waltham, MA, USA) using Qubit dsDNA BR Assay Kit (Thermo Fisher Scientific). Libraries were prepared using TargetRich™ Hereditary Cancer Panel (Kailos Genetics®, Huntsville, AL, United States). Sequencing was performed using Illumina NextSeq 500 system.
Data source location	Faculty of Medicine, Universitas Indonesia, Jakarta, Indonesia
Data accessibility	Raw data (FASTQ) files have been deposited to National Center for Biotechnology Information (NCBI), https://www.ncbi.nlm.nih.gov/, under BioProject database: https://www.ncbi.nlm.nih.gov/bioproject/606794, BioSample database: https://www.ncbi.nlm.nih.gov/biosample?Db=biosample&DbFrom=bioproject&Cmd=Link&LinkName=bioproject_biosample&LinkReadableName=BioSample&ordinalpos=1&IdsFromResult=606794 and SRA database: with accession number: SRR11774092–SRR11774115 (https://www.ncbi.nlm.nih.gov/sra?linkname=bioproject_sra_all&from_uid=606794).

Importance of the data

• Provides information about breast cancer related genes
• Provides novel insights regarding breast cancer development to clinicians and subjects
• Helps to reduce morbidity and mortality via targeted risk management options

## 1 Data description

Cancer is associated with the accumulation of various somatic mutations, structural variants, epigenetic factors, and copy number alterations that occur in a pre-disposed genetic background including hereditary cancers. Advances in sequencing technologies and computational tool development have enabled the implementation of whole‐genome sequencing (WGS) in routine clinical settings, thereby supporting the clinical relevance of genomics with cancer medicine. Precision oncology is a novel approach that directs the clinician to the targeted drug, which is presumed to be effective, after examining the tumour and patient genomes [1].

In this study, we present the data of targeted genome sequence from 24 young women breast cancer patients admitted to Cipto Mangunkusumo National Hospital, Jakarta. Concentration of double-stranded DNA was quantified using Qubit 3.0 (Table 1). Library preparations were set-up using TargetRich™ Hereditary Cancer Panel (Kailos Genetics®). The sequencing process was carried out using Illumina NextSeq 500 and produced 2 × 150 bp paired-end libraries from the sequencing runs (Table 2).

**Table 1:** Double-stranded DNA concentration.

Sample	Qubit 3.0 (C) (μg/mL)
BRCA_35_CMNH_17	45.1
BRCA_36_CMNH_17	52
BRCA_38_CMNH_17	42.7
BRCA_39_CMNH_17	51.6
BRCA_41_CMNH_17	44
BRCA_42_CMNH_17	39.3
BRCA_44_CMNH_17	47.2
BRCA_45_CMNH_17	56.9
BRCA_46_CMNH_17	46.4
BRCA_48_CMNH_17	53.1
BRCA_49_CMNH_17	50.3
BRCA_50_CMNH_17	40.5
BRCA_52_CMNH_17	35.7
BRCA_62_CMNH_17	53.6
BRCA_63_CMNH_17	48.6
BRCA_65_CMNH_17	47.3
BRCA_66_CMNH_17	52.2
BRCA_67_CMNH_17	57.6
BRCA_68_CMNH_17	47
BRCA_70_CMNH_17	41.8
BRCA_71_CMNH_17	37.3
BRCA_72_CMNH_17	47.6
BRCA_73_CMNH_17	52.4
BRCA_75_CMNH_17	51

**Table 2:** Descriptive information of raw sequencing data for targeted genome sequencing.

Sample	BioSample accession number	SRA accession number	Total raw reads	Total raw bases (base-pairs)	Q30 (%)	GC content (%)
BRCA_35_CMNH_17	SAMN14883639	SRR11774094	1,335,991	377,349,417	90.0	39.3
BRCA_36_CMNH_17	SAMN14883641	SRR11774093	1,359,247	389,345,332	87.5	40.0
BRCA_38_CMNH_17	SAMN14883672	SRR11774106	1,270,966	365,237,863	86.2	40.3
BRCA_39_CMNH_17	SAMN14883913	SRR11774101	1,646,642	473,677,411	86.7	40.0
BRCA_41_CMNH_17	SAMN14883916	SRR11774100	1,443,000	415,153,364	86.5	40.0
BRCA_42_CMNH_17	SAMN14883929	SRR11774099	1,346,075	380,618,491	90.2	39.0
BRCA_44_CMNH_17	SAMN14883930	SRR11774098	1,316,741	371,918,891	90.0	40.0
BRCA_45_CMNH_17	SAMN14883931	SRR11774097	1,410,307	405,715,603	87.1	40.0
BRCA_46_CMNH_17	SAMN14883935	SRR11774096	1,391,837	385,392,112	79.2	40.0
BRCA_48_CMNH_17	SAMN14883936	SRR11774095	1,283,974	367,644,572	74.4	40.5
BRCA_49_CMNH_17	SAMN14883938	SRR11774092	2,073,069	560,176,786	77.3	40.1
BRCA_50_CMNH_17	SAMN14883940	SRR11774115	1,392,202	400,365,006	87.0	40.0
BRCA_52_CMNH_17	SAMN14883941	SRR11774114	1,885,957	542,180,051	76.9	40.0
BRCA_62_CMNH_17	SAMN14883942	SRR11774113	1,952,838	540,506,657	82.5	40.0
BRCA_63_CMNH_17	SAMN14883946	SRR11774112	1,399,969	394,781,354	89.5	40.0
BRCA_65_CMNH_17	SAMN14883947	SRR11774111	1,349,591	381,316,421	90.2	39.4
BRCA_66_CMNH_17	SAMN14883948	SRR11774110	1,437,684	413,474,941	86.8	40.0
BRCA_67_CMNH_17	SAMN14883949	SRR11774109	1,339,951	385,231,908	87.1	40.0
BRCA_68_CMNH_17	SAMN14883950	SRR11774108	1,407,335	397,527,460	89.9	39.4
BRCA_70_CMNH_17	SAMN14883951	SRR11774107	1,361,857	385,023,784	90.2	40.0
BRCA_71_CMNH_17	SAMN14883968	SRR11774105	1,522,138	436,958,491	86.3	40.9
BRCA_72_CMNH_17	SAMN14883969	SRR11774104	1,327,112	381,359,342	86.1	40.9
BRCA_73_CMNH_17	SAMN14883970	SRR11774103	2,133,736	613,933,797	86.8	40.0
BRCA_75_CMNH_17	SAMN14883971	SRR11774102	1,478,419	425,334,041	86.5	40.0

FASTQ raw data files have been deposited to the NCBI database under BioProject database: PRJNA606794 (https://www.ncbi.nlm.nih.gov/bioproject/?term=PRJNA606794), BioSample database: https://www.ncbi.nlm.nih.gov/biosample?Db=biosample&DbFrom=bioproject&Cmd=Link&LinkName=bioproject_biosample&LinkReadableName=BioSample&ordinalpos=1&IdsFromResult=606794, and Sequence Read Archive (SRA) accession numbers: SRR11774092–SRR11774115. These data will be useful in analysing the genome markers in breast cancer, and could predict the effective treatments related to their mutation. Methods of sample collection, DNA isolation, library preparation, and sequencing are presented in the following section.

## 2 Experimental design, materials and methods

### 2.1 Sample collection and DNA isolation

Blood samples were collected from 24 young women breast cancer patients. Purified DNA was extracted from the blood buffy coat (it has been recommended to use buffy coat fragments as DNA source [2]) using reagents from the QIAamp DNA Mini Kit® (Qiagen Sciences), as per the manufacturer's recommendation. Double-stranded DNA concentration was quantified using Qubit® 3.0 Fluorometer (Thermo Fisher Scientific) using Qubit dsDNA BR Assay Kit (Thermo Fisher Scientific). Table 1 provides information regarding the concentration of double-stranded DNA of the isolates.

### 2.2 Library preparation

DNA libraries were prepared using TargetRich™ Hereditary Cancer Panel (Kailos Genetics®, Huntsville, AL, United States). Moreover, we used TargetRich™ UMI/Index Adapter Plate (Kailos Genetics®) in patch ligation step. The libraries construction included following steps:

1) Annealing of guide oligonucleotides and restriction digest

About 100 ng of each genomic DNA sample was used as an input and mixed with nuclease-free water and Annealing-Digest Master Mix. The samples were centrifuged briefly to collect the entire liquid at the bottom of the tubes. Thereafter, restriction enzyme was added to each solution and digestion was performed using Thermal Cycler.

2) Patch ligation

TargetRich™ UMI/Index Adapter Plate was added into the aforementioned solution, and DNA ligase was added to each sample. Thermal cycler was used to complete the process.

3) Enzymatic clean-up

Enzymatic Clean-up Master Mix was used to clean the chemical waste from the product used in previous steps.

4) On-bead purification

AMPure® XP beads were used for DNA purification and were added to the mix solution. The cleared solution was discarded, and freshly prepared 70% ethanol was added to it. Without disturbing the beads, ethanol was removed and the beads were air-dried to remove all the traces of ethanol. The DNA on beads was separated by re-suspending the beads in nuclease-free water.

5) PCR amplification

The barcoded-DNA was amplified using combination of Universal PCR Master Mix and DNA polymerase on Thermal Cycler.

6) On-bead purification

The DNA libraries were purified using AMPure® XP beads to remove the chemical waste from the products used in the previous steps [3].

### 2.3 Targeted genome sequencing and data

The DNA libraries were mixed with TargetRich™ UMI/Index Adapter Plate (Kailos Genetics®, Huntsville, AL, United States) for sample barcoding, multiplex sequencing, and tagging of individual captured DNA molecules. The barcoded DNA libraries were sequenced using Illumina NextSeq 500 platform, according to the following steps: 1) preparing the library/PhiX mix; 2) denaturing the library/PhiX mix; 3) diluting the denaturated library/PhiX mix with HT-1 buffer; 4) loading the libraries onto NextSeq 500 reagent cartridge and 5) setting up the sequencing run [4], [5]. The sequencing run produced 2 × 150 bp paired-end libraries (Table 2). The data sequences were deposited to the SRA under the BioProject accession number PRJNA606794. Total raw reads were obtained using FastQC software [6], and the total raw bases and percentage of Q30 were evaluated using q30 python scripts. [7]

## Ethics statements

This research was approved by the Faculty of Medicine Universitas Indonesia Ethical Committee (approval number: 958/UN2.F1/ETIK/2017). Informed consent was obtained from all patients involved in the experiments.

## Declaration of Competing Interest

The authors declare that they have no known competing financial interests or personal relationships which have, or could be perceived to have, influenced the work reported in this article.

## Appendix Supplementary materials

Image, application 1
